# Population Genetics and Signatures of Selection in Early Neolithic European Farmers

**DOI:** 10.1093/molbev/msac108

**Published:** 2022-05-17

**Authors:** Ainash Childebayeva, Adam Benjamin Rohrlach, Rodrigo Barquera, Maïté Rivollat, Franziska Aron, András Szolek, Oliver Kohlbacher, Nicole Nicklisch, Kurt W. Alt, Detlef Gronenborn, Harald Meller, Susanne Friederich, Kay Prüfer, Marie-France Deguilloux, Johannes Krause, Wolfgang Haak

**Affiliations:** Archaeogenetics Department, Max Planck Institute for the Science of Human History, Kahlaische Straße 10, D-07745 Jena, Germany; Archaeogenetics Department, Max Planck Institute for Evolutionary Anthropology, Deutscher Platz 6, D-04103 Leipzig, Germany; Archaeogenetics Department, Max Planck Institute for the Science of Human History, Kahlaische Straße 10, D-07745 Jena, Germany; Archaeogenetics Department, Max Planck Institute for Evolutionary Anthropology, Deutscher Platz 6, D-04103 Leipzig, Germany; ARC Centre of Excellence for Mathematical and Statistical Frontiers, School of Mathematical Sciences, The University of Adelaide, Adelaide, Australia; Archaeogenetics Department, Max Planck Institute for the Science of Human History, Kahlaische Straße 10, D-07745 Jena, Germany; Archaeogenetics Department, Max Planck Institute for Evolutionary Anthropology, Deutscher Platz 6, D-04103 Leipzig, Germany; Archaeogenetics Department, Max Planck Institute for the Science of Human History, Kahlaische Straße 10, D-07745 Jena, Germany; Université de Bordeaux, CNRS, PACEA-UMR 5199, 33615 Pessac, France; Archaeogenetics Department, Max Planck Institute for the Science of Human History, Kahlaische Straße 10, D-07745 Jena, Germany; Applied Bioinformatics, Dept. of Computer Science, University of Tübingen, Tübingen, Germany; Department of Immunology, Interfaculty Institute for Cell Biology, University of Tübingen, Tübingen, Germany; Applied Bioinformatics, Dept. of Computer Science, University of Tübingen, Tübingen, Germany; Institute for Bioinformatics and Medical Informatics, University of Tübingen, Tübingen, Germany; Translational Bioinformatics, University Hospital Tübingen, Tübingen, Germany; Biomolecular Interactions, Max Planck Institute for Developmental Biology, Tübingen, Germany; Center of Natural and Cultural Human History, Danube Private University, Krems-Stein, Austria; State Office for Heritage Management and Archaeology Saxony-Anhalt - State Museum of Prehistory, Halle (Saale), Germany; Center of Natural and Cultural Human History, Danube Private University, Krems-Stein, Austria; State Office for Heritage Management and Archaeology Saxony-Anhalt - State Museum of Prehistory, Halle (Saale), Germany; Römisch-Germanisches Zentralmuseum, Leibniz Research Institute for Archaeology, Ernst-Ludwig-Platz 2, 55116 Mainz, Germany; State Office for Heritage Management and Archaeology Saxony-Anhalt - State Museum of Prehistory, Halle (Saale), Germany; State Office for Heritage Management and Archaeology Saxony-Anhalt - State Museum of Prehistory, Halle (Saale), Germany; Archaeogenetics Department, Max Planck Institute for the Science of Human History, Kahlaische Straße 10, D-07745 Jena, Germany; Archaeogenetics Department, Max Planck Institute for Evolutionary Anthropology, Deutscher Platz 6, D-04103 Leipzig, Germany; Université de Bordeaux, CNRS, PACEA-UMR 5199, 33615 Pessac, France; Archaeogenetics Department, Max Planck Institute for the Science of Human History, Kahlaische Straße 10, D-07745 Jena, Germany; Archaeogenetics Department, Max Planck Institute for Evolutionary Anthropology, Deutscher Platz 6, D-04103 Leipzig, Germany; Archaeogenetics Department, Max Planck Institute for the Science of Human History, Kahlaische Straße 10, D-07745 Jena, Germany; Archaeogenetics Department, Max Planck Institute for Evolutionary Anthropology, Deutscher Platz 6, D-04103 Leipzig, Germany

**Keywords:** selection, neolithization, ancient DNA, Linear Pottery culture

## Abstract

Human expansion in the course of the Neolithic transition in western Eurasia has been one of the major topics in ancient DNA research in the last 10 years. Multiple studies have shown that the spread of agriculture and animal husbandry from the Near East across Europe was accompanied by large-scale human expansions. Moreover, changes in subsistence and migration associated with the Neolithic transition have been hypothesized to involve genetic adaptation. Here, we present high quality genome-wide data from the Linear Pottery Culture site Derenburg-Meerenstieg II (DER) (*N* = 32 individuals) in Central Germany. Population genetic analyses show that the DER individuals carried predominantly Anatolian Neolithic-like ancestry and a very limited degree of local hunter-gatherer admixture, similar to other early European farmers. Increasing the Linear Pottery culture cohort size to ∼100 individuals allowed us to perform various frequency- and haplotype-based analyses to investigate signatures of selection associated with changes following the adoption of the Neolithic lifestyle. In addition, we developed a new method called *Admixture-informed Maximum-likelihood Estimation for Selection Scans* that allowed us test for selection signatures in an admixture-aware fashion. Focusing on the intersection of results from these selection scans, we identified various loci associated with immune function (*JAK1*, HLA-DQB1) and metabolism (*LMF1, LEPR, SORBS1*), as well as skin color (*SLC24A5, CD82*) and folate synthesis (*MTHFR*, *NBPF3)*. Our findings shed light on the evolutionary pressures, such as infectious disease and changing diet, that were faced by the early farmers of Western Eurasia.

## Introduction

The Neolithic transition, a shift from a foraging to a farming-based subsistence, marks one of the most substantial social, ecological, and economic transformations in the prehistory of West Eurasia. The process of neolithization has interested and fascinated archaeologists, anthropologists, demographers, epidemiologists, specialists in evolutionary medicine, and the general public for more than a 100 years (Lubbock 1866; Dawkins 1894; Bogucki 1999; Johnson and Earle 2000; Chamberlain 2006; Bocquet-Appel and Bar-Yosef 2008). In retrospect, the Neolithic was a transformative period in which a mostly sedentary lifestyle, domestication of plants and animals, and the associated relative independence from nature with reliance on sustained food supplies came into being. A manufacturing economy with good yields, surplus, and stockpiling caused an increase in fertility, with earlier age at weaning and subsequent shorter birth intervals which brought about an exponential population increase (Deevey 1960; Bocquet-Appel and Bar-Yosef 2008). The negative effects of neolithization manifested in an increased prevalence and transmission of infectious, metabolic, and nutritional deficiency diseases, favored by sedentary lifestyles, growing populations, and close contact with domesticated animals (Barrett et al. 1998; Cordain 1999; Harper and Armelagos 2010; Gering et al. 2019).

Archaeological research has shown that agriculture and animal husbandry originated in the Near East around 10,000 BCE (Zeder 2011), from where these practices spread to North Africa, Europe, and South Asia. With the help of ancient DNA (aDNA) studies, it was shown that the spread of agriculture across Europe was not a transfer of ideas and technologies but was instead mediated by the expansion of the early farmers (EFs), that is, through demic diffusion (Haak et al. 2010, 2015; Gamba et al. 2014; Lazaridis et al. 2014; Skoglund et al. 2014, 2012; Mathieson et al. 2015; Hofmanová et al. 2016). By ∼6,000 BCE, sedentary farming was established in the Aegean (Horejs et al. 2015), southeastern Europe and the Carpathian Basin (Bánffy 2019), and direct connections have been established between the early European farmers (EEFs) from Neolithic Hungary and Greece and the EFs from western Anatolia (Mathieson et al. 2015; Hofmanová et al. 2016; Lazaridis et al. 2016; Lipson et al. 2017). Confirming previous archaeological findings (Zvelebil 2001; Gronenborn 2014), genetic studies have reported evidence for two routes of EF expansion from the Near East: the inland Central European route; and the coastal (Mediterranean) route to western Europe, Iberia (Olalde et al. 2015; Brunel et al. 2020; Rivollat et al. 2020), and eventually the Atlantic Archipelago (Cassidy et al. 2016; Brace et al. 2019; Rohrlach et al. 2021).

The Linear Pottery Culture (or LBK after German *Linearbandkeramik*) is associated with early accounts of farming in Neolithic Europe (Zvelebil 2004; Bánffy 2019; Petrasch 2020). The LBK culture originated in the western Carpathian Basin in today’s Hungary and Slovakia between 5500 and 5400 BCE and spread across the European loess plains to the Paris Basin in Western Europe (Bickle 2009) and Ukraine in eastern Europe. Archaeologically, LBK emerged from interactions between Starčevo-Körös-Criș complex farmers and local Mesolithic hunter-gatherers (HGs) (Gronenborn 2007). Numerous LBK sites have been found in Central Germany, particularly in the Mittelelbe-Saale (MES) region, a biogeographical region that was attractive to the early Neolithic farmers due to its fertile soils, waterways, and adequate levels of precipitation.

The emergence of agriculture in Western Eurasia has been linked to new environmental and cultural pressures. During this time, HG groups in the area of the Fertile Crescent transitioned to a more sedentary lifestyle associated with increased population sizes, a change in diet and, importantly, increased exposure to infectious diseases from animals, for example, *Salmonella enterica* (Armelagos et al. 1991; Barrett et al. 1998; Harper and Armelagos 2010; Key et al. 2020). Successful settlement in new geographic regions, such as central Europe, would have required rapid adaptation to new environmental, economic, and social conditions. Lower meat consumption and increased caries lesions in teeth indicative of cereal consumption have been reported for the early Neolithic compared to later time periods (Nicklisch et al. 2016; Münster et al. 2018). Moreover, increased prevalence of *cribra orbitalia* and porotic hyperostosis, which indicate either a significant burden of infectious diseases, low quality diet, or a combination of both (Walker et al. 2009), was found at LBK sites (Ash et al. 2016; Nicklisch 2017). A previous study of selection in Europe, using aDNA, in general reported evidence of selection for light skin color, infectious disease resistance, and fatty acid metabolism, among other signals (Mathieson et al. 2015). In addition, Neolithic Aegeans have been shown to carry alleles associated with reduced skin pigmentation and type 2 diabetes susceptibility, and a number of inflammatory disease-associated loci (Hofmanová et al. 2016). Taken together, early Neolithic farmers likely faced selective pressures from the increased exposure to pathogens and the change in diet.

**Fig. 1.**
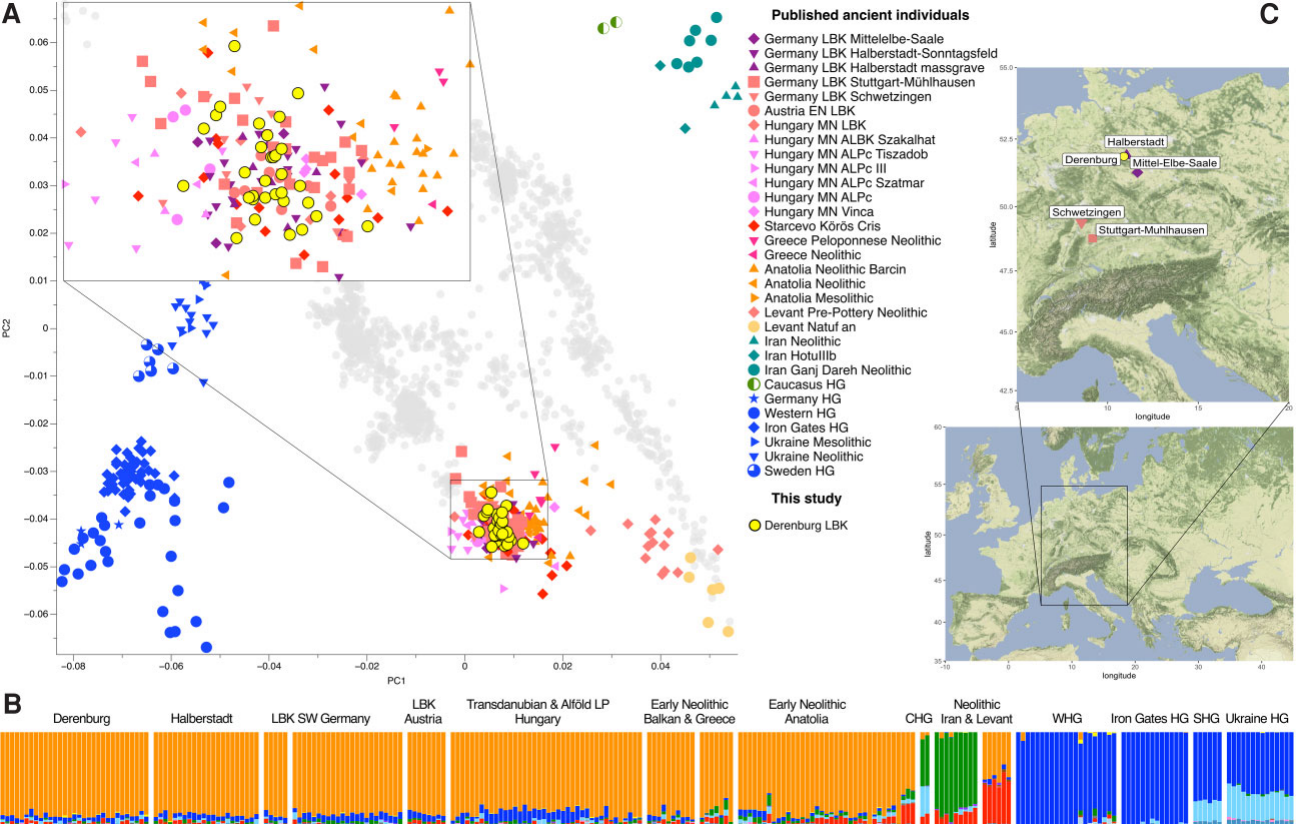
Population genetic analyses of LBK-associated individuals from Derenburg. (*A*) PCA of DER individuals and relevant published ancient groups, projected on West Eurasian genetic variation. (*B*) Unsupervised ADMIXTURE results (*k* = 12) of a subset of individuals shown in panel A. (*C*) Map of Europe and zoom in on LBK sites in today’s Germany. Map tiles in panel (*C*) by Stamen Design, under CC BY 3.0. Data by OpenStreetMap, under ODbL.

Here, we report new genome-wide data from human remains associated with an LBK burial site Derenburg-Meerenstieg II (Wernigerode, Saxony-Anhalt, Germany). The Derenburg (DER) burial ground is located in Saxony-Anhalt, close to the Holtemme tributary of the Bode River, in the northern Harz foreland (fig. 1). Previous studies using stable isotopes have shown that mean adult human values for carbon and nitrogen isotopes were typical for the region and suggested a mixed farming diet including domesticated plant and animal products (Oelze et al. 2011; Münster et al. 2018). A previous study of mitochondrial DNA (mtDNA) lineages from the EEF, including the individuals in this study, showed that the mtDNA haplogroups of LBK individuals and their frequency distribution is more similar to the present-day population of Anatolia and the Near East (Haak et al. 2010). This indirectly argued for demic diffusion from Anatolia to Europe and a degree of genetic continuity between the two regions, which was later confirmed using genome-wide studies (Mathieson et al. 2015; Hofmanová et al. 2016; Lazaridis et al. 2016; Lipson et al. 2017; Rivollat et al. 2020).

One of our aims was to provide an updated genomic portrait of DER in the context of genome-wide variation of the EEF. We explored the genomic ancestry of LBK individuals comparing them to other EEF and Anatolian EFs, as well as local European Western Hunter-Gatherers (WHGs) on a broader scale. We also investigated the intra-site biological relatedness and explored the demography of LBK groups by comparing DER individuals to previously published individuals from nearby sites, such as Halberstadt (only ∼10–15 km apart), and other LBK sites from Germany, Poland, Austria, and Hungary (Lipson et al. 2017; Mathieson et al. 2018; Rivollat et al. 2020).

## New Approaches

Leveraging the substantially increased cohort size of the EEFs, we performed various scans of selection to determine specific genes and pathways that were under selection in LBK individuals, as well as the EFs in general. To our knowledge, genetic adaptation associated with the Neolithic lifestyle has not been formally tested using aDNA. We performed allele frequency—[LSBL and our own newly developed method called *Admixture-informed Maximum-likelihood Estimation for Selection Scans* (AIMLESSs)] and haplotype-based selection scan (XP-EHH), and analyses of the HLA class I and II alleles, to investigate whether the LBK population was under an evolutionary pressure as a result of the Neolithization and population migration/expansion. Moreover, we performed selection tests comparing EF individuals to modern-day African populations and the local HGs to test general adaptation to the Neolithization in Western Eurasia.

The ability to detect true loci that are under selection is based on how well the population structure is accounted for, especially in a test that depends on comparisons between populations. Thus, we developed AIMLESS to test for selection in an ancestry-aware fashion. This method is a modification of the admixture-aware linear model-based test developed by Mathieson et al. (2015); however, our test is specifically designed for two population admixture scenarios. AIMLESS is similar to other likelihood ratio test (LRT)-based tests ssuch as *AdaptMix* (Mendoza-Revilla et al. 2021) and Ohana (Cheng et al. 2022) but does not rely on a priori ancestry estimation and determines the mixture components for the ancestries considered for each single-nucleotide polymorphism (SNP) independently instead of using a genome-wide estimate.

## Results and Discussion

### Data Generation and Authentication

We generated genome-wide SNP capture data for ∼1.24 million variant sites (1,240k SNP array) across the genome (Fu et al. 2015; Mathieson et al. 2015), mitochondrial genome capture (Maricic et al. 2010), Y-chromosome capture (Rohrlach et al. 2021) and 3 Mb immune-capture data (Immel et al. 2021) from *N* = 32 individuals. In addition, we sequenced the complete genomes of two DER individuals (DER002 2.26X and DER009 3.07X), and a local HG individual from Bad Dürrenberg, Germany (BDB001 13x), for which genome-wide SNP capture data was previously reported (Rivollat et al. 2020). A subset of DER individuals (*N* = 10) was radiocarbon dated to ca. 5,300–4,800 cal. BCE (supplementary table S1, Supplementary Material online) or ∼7,000 years before present (BP), which is consistent with the younger phase of the LBK period in Germany (Gronenborn et al. 2014).

The authenticity of human aDNA from the DER individuals was confirmed using MapDamage (Ginolhac et al. 2011), wherein all aDNA fragments were shown to have deamination patterns consistent with degradation over time, and unambiguous sex determination. Male samples were checked for X chromosome contamination from modern sources, using ANGSD (Korneliussen et al. 2014). Mitochondrial DNA contamination was estimated using Schmutzi (Renaud et al. 2015) and contamMix (Fu et al. 2013). No significant contamination (>5%) from modern sources was determined in the DER cohort (supplementary table S2, Supplementary Material online). Based on the genetic sex determination, we identified 18 females and 14 males (54.5% female).

### Genome-Wide Data Analysis

We genotyped the 1,240k SNP positions using pileupCaller (https://github.com/stschiff/sequenceTools/tree/master/src-pileupCaller) obtaining pseudohaploid calls for each variant position on the capture. We merged the 1,240k SNPs with the modern-day Human Origins data set, as well as relevant ancient samples (https://reich.hms.harvard.edu/allen-ancient-dna-resource-aadr-downloadable-genotypes-present-day-and-ancient-dna-data, version 44.3), to perform a principal component analysis (PCA). Owing to the incomplete nature of the ancient data, ancient individuals were projected onto the genetic variation of 1363 modern West Eurasians (fig. 1*A*). Based on the position in PC space, we found that the DER individuals fall together with published LBK individuals, as well as contemporaneous early Neolithic individuals from southeastern Europe and western Anatolia (fig. 1*A*). We then performed an unsupervised ADMIXTURE analysis and found that at *K* = 12 DER represent a mixture of an ancestry component maximized in Anatolian Neolithic individuals and a smaller proportion of ancestry component maximized in WHGs (fig. 1*B*).

**Fig. 2.**
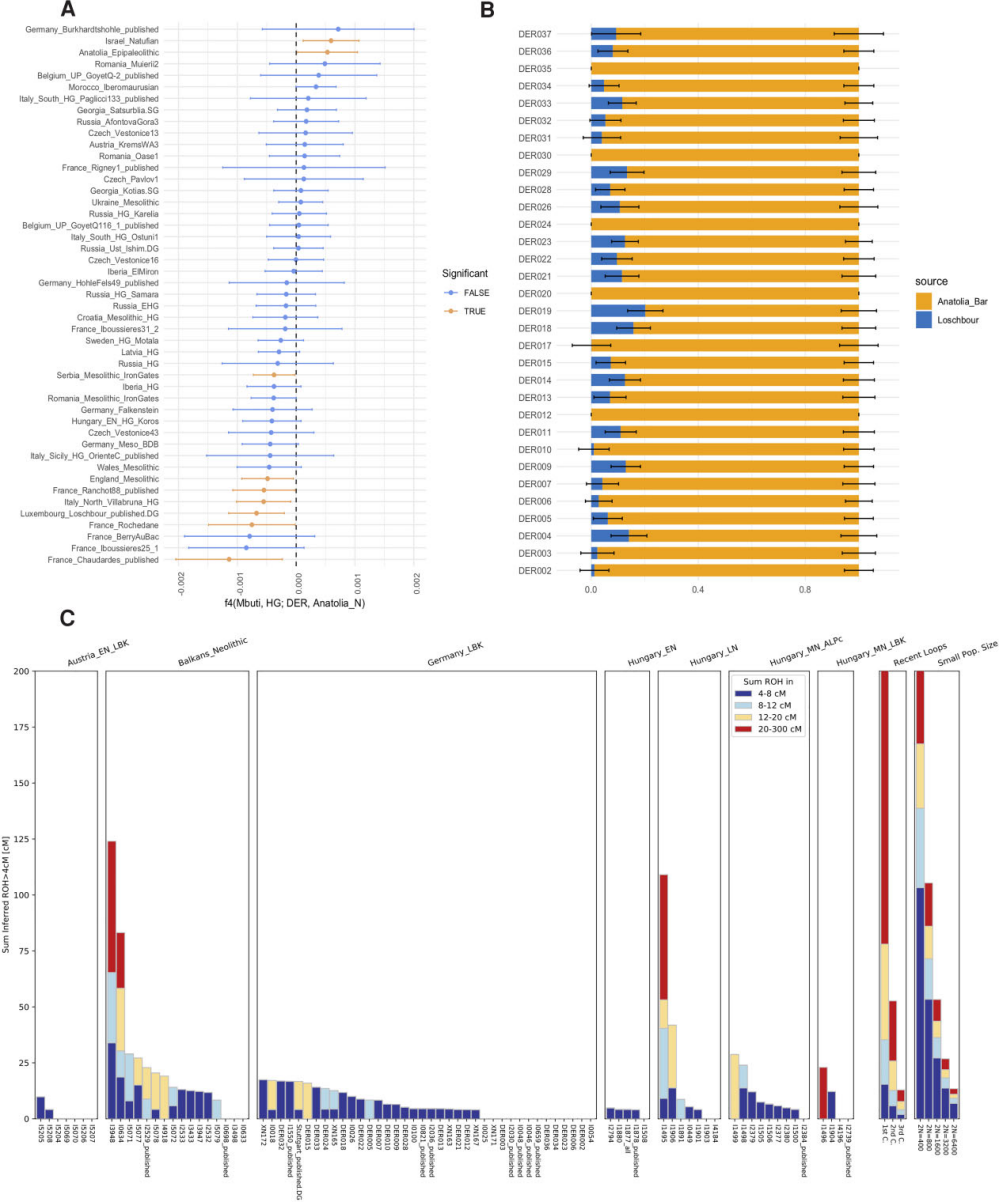
Hunter-Gatherer admixture modeling. (*A*) F4 analysis of the form *f4*(Mbuti, European_HG; DER, Anatolia_N). Each hunter-gatherer individual tested is represented on the *y*-axis. Tests with *z*-scores > 3 are shown in orange compared to rest in blue; (*B*) *qpAdm* test results for DER individuals using the Loschbour HG and Anatolia_N_Barcin individuals as admixture sources. All models have *P* values >0.05, which indicates models that cannot be rejected formally in case of *qpAdm*; (*C*) HapROH output for German LBK individuals compared to EEF from Hungary, Austria, and the Balkans. Only samples with more than 400k called 1,240k SNPs were included.

Following the PCA and ADMIXTURE analyses, we calculated various *F*-statistics to formally test the ancestry components identified in the admixture analysis and inferred from the position on the PCA plot. We first performed an f4-test of the form f4(Mbuti, HG; DER, Anatolia_N) to test whether DER individuals carried additional HG ancestry compared with Anatolia_N (fig. 2*A*). Significantly negative f4-values (|*Z*| > 3) for models in which England_Mesolithic, Villabruna, Loschbour HG, France_Rochedane, and France_Chaudardes were used as HG, indicated excess WHG ancestry in DER. We also obtained a significantly negative f4-value for the Iron Gates HGs (Mathieson et al. 2018).

In a follow-up f4-analysis of the form f4(Mbuti, HG; EEF, Anatolia_N) we iterated through a number of EEF groups and tested the following two HG sources specifically: Loschbour HG (WHG) and Iron Gates HGs (supplementary fig. S1, Supplementary Material online). In addition, we tested BDB001 (Rivollat et al. 2020) as a local HG ancestry proxy from the MES region. The DER individuals showed significantly negative f4-results suggesting more HG ancestry compared to Anatolia_N in the first two cases (Loschbour HG and Iron Gates), but not for the local HG proxy BDB001. This suggests that DER individuals had received additional HG admixture upon their expansion into Europe, but not HG ancestry immediately related to the local BDB001 source. When comparing all LBK groups, many were cladal with Anatolia_N, indicating a rather swift expansion with only occasional contribution of additional (and local) HG ancestry.

In turn, when comparing the HG sources to each other, we could not identify which source contributed the most to DER (supplementary fig. S2, Supplementary Material online). However, we did see an increased affinity of DER to the Loschbour HG representing the WHG ancestry, compared with Iron Gates HGs who have additional eastern HG ancestry (supplementary fig. S2, Supplementary Material online) (Mathieson et al. 2018).

We then used qpAdm to quantify the relative contribution of HG ancestry represented by the Loschbour HG and the Anatolia_N ancestry represented by Neolithic individuals from Barcin, western Anatolia (Mathieson et al. 2015). When modelled individually, we found that DER individuals had between 0% and 20% (SE 6.6%) European HG ancestry (fig. 2*B*, supplementary table S3, Supplementary Material online). When modeled as a group, we found that on average DER individuals had ∼7% HG and ∼93% western Anatolian Neolithic ancestries, indicating limited HG contribution (fig. 2*C*).

Using the same set of sources (Anatolia_N and Loschbour HG), we estimated the time of admixture between the Anatolian farmers and WHGs using DATES v. 753 (Moorjani et al. 2016) to have occurred 24.286 ± 2.784 generations ago (supplementary fig. S3, Supplementary Material online), or ∼680 calendar years before the time of the DER individuals assuming a generation time of 28 years (Fenner 2005), which falls at the beginning of the Starčevo-Körös-Criș complex (∼6,200–5,200 BCE) of the southeastern European Neolithic (Porčić *et al*. 2020).

According to both the model-free ADMIXTURE and the qpAdm results with a defined set of sources, we can show very limited HG ancestry influx in the DER LBK group in addition to the HG ancestry already carried by Anatolian farmers. This suggests minimal interaction between European HG and expanding early LBK farmers from southeastern Europe during the Neolithic, corroborating findings by other studies (Lipson et al. 2017; Mathieson et al. 2018; Rivollat et al. 2020).

### Uniparentally Inherited Markers

Mitochondrial DNA and Y-chromosome haplogroups of the DER individuals are generally consistent with the genetic variation reported from early Neolithic farmers (supplementary table S1, Supplementary Material online) (Brandt et al. 2013; Szécsényi-Nagy et al. 2015), and the mitochondrial genomes match the HVR-I haplotypes reported earlier (Haak et al. 2010). We primarily found G2a2 and H2 Y haplogroups associated with Neolithic farmers (Lacan et al. 2011; Haak et al. 2015; Rivollat et al. 2020; Rohrlach et al. 2021). We also observed one male with the Y-chromosome I haplotype that is generally common among European HGs and therefore considered as a signal of male HG ancestry contribution but also reported in lower frequency from Iberian and French Neolithic individuals (Lipson et al. 2017; Rivollat et al. 2020).

### Biological Relatedness

Based on the analysis of biological relatedness via READ (Monroy Kuhn et al. 2018) and lcMLkin (Lipatov et al. 2015), we identified three first-degree relationships (two-parent offspring, one sibling pair) and second-degree related pairs (supplementary table S1, Supplementary Material online). The mother-daughter pair (DER019 mature female – DER018 infant) was buried in the same grave, whereas the mother-son pair (DER022 mature female – DER011 mature male) was not. Despite the integration of contextual anthropological and archaeological evidence such as age-at-death and uniparentally inherited haplogroups, we were not able to construct the true single pedigree of the 2nd degree pairs, as several alternatives were possible.

We performed Wilcoxon Rank Sum tests on the mean measure of relatedness for each individual to the rest of the group as calculated from READ to see whether there is a general skew towards males or females from the site being more closely related to each other, which would indicate either potential patri- or matrilocality. We compared the pair-wise relatedness of all males to all females (*P* value = 0.34), all adult males to females (*P* value = 0.12), and the subadults (*P* value = 0.35), none of which significantly differed from each other (supplementary fig. S4, Supplementary Material online).

We determined the degree of inbreeding in the DER cohort and compared it with other Neolithic EEF populations using hapROH 0.1a6 (Ringbauer et al. 2021). Based on the analysis of runs of homozygosity (ROH), we found evidence of low levels of background relatedness as indicated by low values for the sum of short ROH segments. Moreover, compared with other EEF populations, we did not see different levels of inbreeding among individuals at DER (fig. 2*E*). In general, we found consistently low levels of inbreeding across all early Neolithic groups in Central Europe, suggesting that EEFs lived in larger groups or groups with an extended mating network, and ultimately stemmed from a source deme with a large effective population size (Ringbauer et al. 2020).

The biological relatedness at DER is similar to other LBK sites from Germany, for example, Halberstadt-Sonntagsfeld, where one first- and two first- or second-degree related pairs were observed of 24 total individuals (Lipson et al. 2017). Considering that the DER burial site is archaeologically a closed find and that the temporal spread of the calibrated years before present (calBP) is around 300 years, 6848.5–7141calBP (supplementary table S1, Supplementary Material online), the few first- and second-degree relationships among a total of 32 genotyped DER individuals suggest that the site was used over a longer period of time and not restricted to (a) particular biological kin group(s).

### LBK Inter-site Comparisons

We further tested the observations on LBK population structure inferred from biological relatedness results using inbreeding coefficients and effective population size comparisons of LBK sites in Germany with other already published EEFs. We used READ (Monroy Kuhn et al. 2018) to test for relatedness between individuals from DER and other LBK sites in Germany, mainly already published data from Halberstadt-Sonntagsfeld (Germany_LBK_HBS, *N* = 29) and Stuttgart-Mühlhausen (Germany_LBK_SMH, *N* = 29) (Lipson et al. 2017; Rivollat et al. 2020). To do this, we first estimated the median relatedness in each German LBK group separately and found them to be comparable with each other (LBK_HBS = 0.24; LBK_SMH = 0.25; LBK_DER = 0.24). We then performed a joint biological relatedness analysis of all German LBK sites using READ and found no evidence of first- or second-degree relatedness between individuals from different sites.

To investigate this further, we estimated the effective population size (*N*_e_) in LBK as implemented in (Fernandes et al. 2021). Using the maximum-likelihood to fit N_e_, we estimated the effective population size to have been ∼5,000 individuals (3,688–6,778 95% CI) for all LBK sites from Germany, which is consistent with effective population size estimates for a relatively large population (Tenesa et al. 2007; Melé et al. 2012). Together with the results from our analysis of the lack of inbreeding and inter-site biological relatedness, this further suggests that the early Neolithic LBK farmers were part of a larger population, did not practice close biologically related mating, and prevented inbreeding. In comparison, based on limited biological relatedness among individuals in intramural burials, it has been suggested that Neolithic Anatolia was not a strictly kin-based society (Pilloud and Larsen 2011; Yaka et al. 2021).

### Phenotypic Analysis

We determined genotype likelihoods for a select number of SNPs associated with genetic adaptation, including lactase persistence, metabolic adaptation, among others, as well as SNPs associated with phenotypic traits as determined by the H-IrisplexS platform (Walsh et al. 2014; Chaitanya et al. 2018) (supplementary fig. S5, Supplementary Material online). Based on the analysis of the SNPs associated with phenotypes, we see the highest frequencies of brown (33%) eye color, intermediate skin color (21%), and brown (21%) hair color. We report these data being fully aware of the fact that the analysis of phenotypic variation can be a contentious issue, due to the fact that it was developed based on modern individuals of predominantly European ancestry, and does not perform as well in admixed individuals and populations outside of Europe (Dembinski and Picard 2014, 2016; Yun et al. 2014). Thus, we note that these data, and specifically eye, skin, and hair color, are frequent requests of museums and other organizations to illustrate reconstruction life histories of past societies.

### HLA Class-I and II Allele Analysis

HLA Class-I and II haplotypes in the DER individuals were analyzed using Optitype (Szolek et al. 2014). Based on the analysis, we report the highest allele frequencies for *HLA-A* to be A*24:02 (0.44) and A*02:01 (0.38), B*51:01 (0.31) and B*27:05 (0.25) for *HLA-B*, and C*02:02 (0.25) and C*15:02 (0.23) for *HLA-C* (supplementary tables S4 and S5, Supplementary Material online). In comparison, Late Neolithic individuals from the Wartberg culture show similar distributions of the HLA class I alleles, with HLA-B*27:05 at a frequency of 23% and HLA-C*02:02 at 17% (Immel et al. 2019), which is higher than the modern frequencies of the two alleles in Europeans being 3.73% (Wu et al. 2021) and 5.6% (UK pop 3 from (Gonzalez-Galarza et al. 2021)), respectively. Interestingly, the two highest frequency *HLA-B* alleles in our sample, HLA-B*51:01 and HLA-B*27:05, have been associated with Behçet’s disease susceptibility (Verity et al. 1999; Kirino et al. 2013) and ankylosing spondylitis (AS) (Cauli et al. 2013; International Genetics of Ankylosing Spondylitis Consortium (IGAS) 2013; Chen et al. 2017). Based on the skeletal analysis of the individuals, the carriers of the HLA allele B*27:05 among the DER individuals did not show skeletal signs of AS, even though ∼90% of the AS patients are HLA-B*27^+^ (Brewerton et al. 1973; Schlosstein et al. 1973; Woodrow and Eastmond 1978). Although only 1–5% of the HLA-B*27 positive individuals develop AS in the general population (van der Linden et al. 1983; Reveille et al. 2010), it is recognized as one of the most important monogenic associations for this disease known to date (van der Linden et al. 1983; Reveille et al. 2010; Karnes et al. 2017). It is possible that some of the carriers in our sample could have still developed the condition later in life (Feldtkeller et al. 2003). The most frequent HLA class II alleles are HLA-DRB1*11:01 (0.42), -DRB3*02:02 (0.48), -DQA1*05:05 (0.44), and -DQB1*03:01 (0.52) (supplementary tables S4 and S5, Supplementary Material online). All these alleles show similar frequencies as they are all part of the same HLA class II haplotype (43.75%), which was reported in relatively high frequencies in present-day central European populations such as Czech Republic (15.30%) (Zajacova et al. 2016), but also in Nganasan from Siberia (18.80%) (Uinuk-Ool et al. 2004).

### Selection Scans

The transition from hunting and gathering to agriculture and animal husbandry in the Neolithic has been linked to new selective pressures due to an increased pathogen burden (Armelagos et al. 1991; Key et al. 2020) and a shift to a cereal-based diet together with decreased nutritional diversity (Luca et al. 2010). We performed two types of selection scans (frequency- and haplotype-based) to detect signatures of selection associated with Neolithization in the newly generated DER cohort together with already published data. We first describe the settings and cut-offs used in each analysis and then address the intersection of the results in a joint discussion.

We first used a locus-specific branch length (LSBL) test for selection (Shriver et al. 2004). The LSBL test is based on the pair-wise genetic distances between three populations, wherein each SNP is considered independently. SNPs with the longest Fst branches in one population compared to the other two are considered as potential selection candidates. We performed three different LSBL tests to determine whether the transition from hunting and gathering to an agricultural lifestyle and the population expansion from the Near East was associated with adaptation by natural selection to new environments in higher latitudes of Europe and to the Neolithic way of life in general.

First, we compared three cohorts consisting of LBK (*N* = 97), Neolithic individuals from western Anatolia (*N* = 37), and autochthonous HGs as local control group (*N* = 86), (see supplementary fig. S6, Supplementary Material online for the PCA with the individuals used in our cohorts, and supplementary table S6, Supplementary Material online for the list of the individuals). We chose to compare the LBK to the Anatolian farmers because these populations are known to be closely related based on our PCA and Admixture analyses, as well as previous studies (Mathieson et al. 2015; Lazaridis et al. 2016). SNPs with the largest pair-wise Fst values between Anatolian farmers and the LBK, as well as between LBK and HGs were prioritized, since these SNPs could indicate selection for the new environmental or lifestyle conditions associated with the expansion into and across Europe, and ongoing Neolithization, respectively. Based on the LSBL analysis, we prioritized *N* = 9549 genetic loci under the empirical quantile <0.01 (top 1%) that was calculated based on the distribution of the LSBL statistic following previous studies (Bigham et al. 2010).

Second, to test for selection since the deeper split of LBK and Anatolia_N from WHG, we merged the ancient farmer populations (LBK and Anatolia) into a single EF group, and compared it with WHG, and modern Esan (ESN) individuals from the 1KG data set (The 1000 Genomes Project Consortium 2015) as a non-European farming group comparison. In a third analogous LSBL analysis, we also compared EF to WHG and an African HG cohort represented by Biaka and Mbuti from the SGDP and HGDP data sets, respectively (Mallick et al. 2016; Bergström et al. 2020). During this deeper time frame, we expect the Neolithization in Western Eurasia to have had a substantial effect due to the change in subsistence and increased population densities and disease susceptibility.

We also performed an ancestry-aware selection scan using our newly developed method AIMLESS. For each position on the genome for which we had allele frequencies for all three groups (LBK, Anatolia_N, and WHG), we performed a maximum-likelihood estimation (MLE) of allele frequencies in the LBK based on the observed data (unconstrained model), which we compared with the MLE based on the most likely admixture of Anatolia_N and WHG ancestries (constrained model). We performed a likelihood ratio test comparing two models and determined *P* values based on a χ^2^ distribution with two degrees of freedom and considered loci with *P* value < 1e-8 as significant based on simulation studies (supplementary figs. S7 and S8, Supplementary Material online).

To look for more recent signals of adaptation (Szpiech and Hernandez 2014) to the Neolithic lifestyle through the comparison of LBK to WHG, we also explored a haplotype-based selection test called *Cross Population Extended Haplotype Homozogysity* (XP-EHH), which can detect selective sweeps in one population compared with another (Sabeti et al. 2007). To do so, we first used GLIMPSE (Rubinacci et al. 2021) to impute and phase the genome-wide data from DER and already published LBK individuals (Lazaridis et al. 2014; Mathieson et al. 2015; Lipson et al. 2017; Rivollat et al. 2020), as well as Iron Gates HGs (Mathieson et al. 2018). To understand the coverage cutoff for imputation, we performed a down-sampling experiment, wherein we down-sampled a high-coverage (92×) 1,240k SNP captured LBK individual from Stuttgart Mühlhausen, which was obtained as a result of routinely using this library as positive control in capture experiments. Based on our test of the genotype agreement between the down-sampled and diploid data, we decided on a cutoff of 0.5× (LBK: *N* = 31, Iron Gates: *N* = 27) for the inclusion of the imputed samples in the downstream selection scans with selscan (Szpiech and Hernandez 2014) (supplementary fig. S9, Supplementary Material online). We identified 52,648 sites with the absolute XP-EHH value >2.

Owing to the limitations of missing data inherent in aDNA and to further overcome the potential issue of false positive results based on imputation or factors such as drift and population structure, we prioritized results that were overlapping between XP-EHH, LSBL, and AIMLESS, since it has been shown that a combination of multiple tests is more powerful to detect true selection signals (Grossman et al. 2010). An important caveat is that we used the 1 KG data as modern reference panel (The 1000 Genomes Project Consortium 2015) to impute the ancient data. This is not ideal since imputation with a modern reference can introduce spurious genotypes and make the individuals imputed more similar to the reference panel used. We are also aware that the European gene pool underwent further transformation during the Bronze Age (Allentoft et al. 2015; Haak et al. 2015). This is why the selection results based on imputed data have to be analyzed and interpreted with caution.

As predicted, we identified many SNPs associated with immune function and metabolism among the LSBL top 1% hits from the comparison of LBK versus Anatolia_N and WHG (supplementary table S7, Supplementary Material online). To determine if any biological pathways are over-represented among the top SNPs identified by the LSBL test of LBK versus Anatolia_N and WHG, we used Gowinda (Kofler and Schlotterer 2012) for a gene-set enrichment analysis. We identified 110 significant Gene Ontology (GO) terms after a correction for multiple comparisons using the false discovery rate cutoff of 0.05. Among the significant results, we found 8 of 110 GO terms associated with lipid metabolism (OR: 1.82 compared with all GO terms) (GO0061365: positive regulation of triglyceride lipase activity, GO0051006: positive regulation of lipoprotein lipase activity, GO0060193: positive regulation of lipase activity, GO0015909: long-chain fatty acid transport, GO0015908: fatty acid transport, among others) and 6 out of 110 with the immune system (OR: 0.65 compared with all GO terms) (GO0006911: phagocytosis, GO0002376: immune system process, GO0050900: leukocyte migration, GO0001776: leukocyte homeostasis, and others) (supplementary table S8, Supplementary Material online). We summarized and visualized the GO terms with REVIGO (Supek et al. 2011) using the default parameters (supplementary fig. S10, Supplementary Material online). Among the GO term “superclusters” we identified: “long-chain fatty acid transport”, “leukocyte homeostasis”, “positive regulation of lipoprotein lipase activity”, “immune system process”.

Overall, we found 227 overlapping positions between the three LSBL tests (supplementary tables S9–S11, Supplementary Material online). Notably, those included SNPs associated with *APOM* rs805297 (linked to rheumatoid arthritis and lipid signaling), *BDNF* rs11030104 (BMI), *IGSF21* rs12031938 and *ILDR2* rs10489574 (immune response), *LMF1* rs12933840 and rs8062983 (lipid metabolism), MHC SNPs rs2844482 and rs9277027 (immune function), and *PDE2A* rs11235559 (susceptibility to viral infections) (Fumagalli et al. 2010). Our findings from the LSBL comparisons highlight the immune and lipid metabolism pathways as strong selection candidates. This is not surprising, since immune function adaptation has been shown before in previous analyses of natural selection in Western Europe from the Neolithic to modern time (Mathieson et al. 2015; Chekalin et al. 2019). To our knowledge, this is the first time that the continuous selection on the immunity related pathways is shown with regards to the Neolithic expansion from the Near East to Europe.

Among the SNPs that were identified by the LSBL tests for deeper selection in the early Neolithic farmers, we find loci associated with skin color (*SLC24A5, CD82*) adaptation and folate synthesis (*MTHFR*, *NBPF3*) (supplementary tables S9 and S10, Supplementary Material online). Notably, *SLC24A5,* has been also identified by previous studies of selection in Europeans using aDNA (Mathieson et al. 2015; Ju and Mathieson 2021). Skin color variation is one of the most striking features of human phenotypic diversity. Skin color is highly correlated with latitude and the resulting ultraviolet radiation (UVR) intensity, and populations closer to the equator have darker skin color, whereas the populations located away from the equator have lighter skin color (Jablonski and Chaplin 2000). It has been long hypothesized that skin color variation has evolved as a balance between vitamin D synthesis and folate synthesis, as well as skin cancer protection. On one hand, vitamin D is synthesized upon UVR exposure (Jablonski and Chaplin 2010), and, on the other hand, folate has to be protected from photolysis from UVR (He et al. 2009). Our findings support the idea of coevolution of folate synthesis and lighter pigmentation in the EF, compared to the HGs and the African outgroup populations. Moreover, our study narrows down the timing of selection on *SLC24A5* to after the split between the European HGs and the ancestors of the early Neolithic farmers. Of note, European HGs are characterized by a darker skin color, even though they occupied higher latitudes with low UVR exposure. The current hypothesis is that the diet based on meat/fish served as a sufficient source of vitamin D in higher latitudes for the HGs, similar with what has been shown in the Inuit and other Arctic indigenous populations (Kolahdooz et al. 2013; Schaebel et al. 2015).

We analyzed SNPs that are associated with pigmentation in the ancient populations separately (LBK, Anatolia_N, WHG) (table 1), since we hypothesized that migration to a higher latitude from Anatolia to central Europe could be associated with positive selection for light skin color phenotype. Based on the comparison between LBK and Anatolia_N, we did not see a major shift in skin color SNPs that are involved in determining lighter skin pigmentation, suggesting that this adaptation already happened in Anatolia/the Near East during the transition to a sedentary farming lifestyle.

**Table 1.** SNPs Associated with Pigmentation and Other Phenotypes of Interest.

Gene	Phenotype	SNP	REF	ALT	EFF	LBK	Anatolia	WHG	AFR	EAS	EUR
*FADS1/FADS2*	Fatty acid Metabolism	rs174546	C	T	T	0.70	0.71	0.97	0.02	0.57	0.35
*TYR*	Light skin (WE)	rs10831496	A	G	A	0.28	0.42	0.13	0.17	0.25	0.68
*TYR*	Light skin (WE)	rs1042602	A	C	A	0.17	0	0	0.01	0.00	0.37
*OCA2*	Light skin (EE)	rs1800414	C	T	C	0	0	0	0.01	0.60	0
*OCA2*	Light skin (WE)	rs1800404	C	T	T	0.73	0.85	0.83	0.13	0.39	0.79
*OCA2*	Blue eyes (WE)	rs12913832	G	A	G	0.42	0.30	0.55	0.03	0.00	0.64
*APBA2*	Light skin (WE)	rs4424881	C	T	C	0.90	0.95	0.71	0.10	0.45	0.87
*SLC24A5*	Light Skin (WE)	rs1426654	A	G	A	0.89	1	0.47	0.07	0.01	1.00
*SLC24A5*	Blue eyes (WE)	rs2470102	A	G	A	0.90	1	0.44	0.07	0.25	0.99
*MC1R*	Light skin (EE)	rs2228479	A	G	A	0.03	0	0.08	0.00	0.29	0.07
*MHC*	Immunity	rs2269424	A	G	A	0.73	0.89	0.68	0.01	0.14	0.26

The results from the XP-EHH test confirmed well-known selection signals linked to skin color adaptation in the gene *SLC24A5* (Lamason et al. 2005) (supplementary fig. S11, Supplementary Material online). Selection signature around the *SLC24A5* core SNP rs1426654 (xpehh = 5.13) suggests that the haplotype that is associated with lighter skin color is highly prevalent in the LBK individuals and is also under selection in the Iron Gates HGs. We also identified *FADS1* as a potential selection candidate by the LSBL test comparing LBK individuals to Anatolia_N and WHG, and by XP-EHH for SNP rs174546 (xpehh = −2.05) (supplementary fig. S12, Supplementary Material online). The *FADS1/FADS2* locus has been identified before by previous studies of selection using ancient individuals. It has been shown that FADS was under selection both before and after the Neolithic transition (Ye et al. 2017), with the ancestral allele under selection before (Mathieson 2020) and the derived after (Martiniano et al. 2017).

A novel gene of interest that was also identified by both LSBL and XP-EHH is *LMF1* (fig. 4*A*). LMF1 or lipase maturation factor 1 is involved in the lipase system, and is required for the maturation of the lipoprotein lipase, hepatic lipase, and endothelial lipase [for review see (Péterfy 2012)]. LMF1 activity is associated with levels of triglycerides, and its mutations are linked to hypertriglyceridemia, or the presence of high levels of triglycerides, and lipase deficiency (Péterfy et al. 2007). LMF1 is present at high levels in mice embryos, suggesting a potential, although currently unknown, role in embryonic development (Ehrhardt et al. 2014). Plant-based diets are characterized by a lower lipid content (Yokoyama et al. 2017), and efficient lipid metabolism could have been under selection especially in LBK individuals, who have been shown to have higher rates of caries than individuals in the Late Neolithic, indicative of the reliance on cereals (Nicklisch et al. 2016). Another novel selection candidate is *JAK1* (fig. 4*B*). The JAK-STAT pathway is involved in the tuning of immune response, and is associated with immune disorders (Witalisz-Siepracka et al. 2018). Janus kinases are involved in immune cell signaling, and complete deletions of Jak1 and Jak2 are not compatible with life in mice (Ghoreschi et al. 2009). Polymorphisms in *JAK1* have been linked to blood cell type composition (Astle et al. 2016), white blood cell count (Kichaev et al. 2019).

**Fig. 3.**
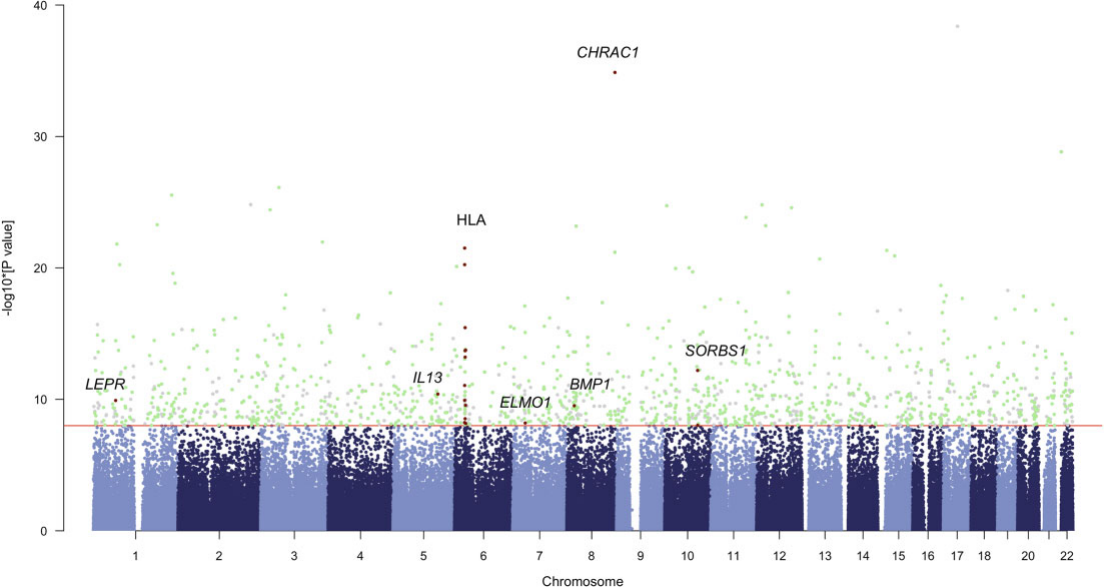
Manhattan plot of the results of the selection scans. Chromosomes are shown on the x axis, whereas the -log_10_(*P* values) from AIMLESS are represented on the *y* axis. Significant loci that are shared between AIMLESS, LSBL (LBK vs. Anatolia_N and HG), and XP-EHH are highlighted in green. Loci that do not overlap between the three tests are shown in gray. The loci related to immunity and metabolic pathways are highlighted in red.

Lastly, we explored all 971 loci that are shared between the three selection tests AIMLESS, XP-EHH and LSBL (LBK vs. Anatolia_N and HGs). These include SNPs associated with *LEPR*, *IL13*, HLA-A, HLA-B, HLA-DQB2, *CHRAC1*, *BMP1*, *SORBS1*, *ELMO1*, and others (supplementary table S12, fig. 3), among which SNPs associated with immune function and metabolism such as *HLA-DQB1*, *SORBS1,* and *LEPR* stood out (supplementary table S12, Supplementary Material online). The HLA-DQ molecule is a major histocompatibility complex (MHC) class II protein, expressed in B lymphocytes, dendritic cells, and macrophages. Diseases that are associated with the genes HLA-DQA1 and -DQB1 coding for HLA-DQ include Celiac Disease (Zubillaga et al. 2002; Megiorni and Pizzuti 2012), autoimmune diseases (Spurkland et al. 1991; Badenhoop et al. 1995), and viral resistance (Huang et al. 2019). Interestingly, *HLA-DQB1* showed a strong signature of balancing selection in regions where Celiac Disease is common (Solberg et al. 2008; Sams and Hawks 2014), indicating its involvement in the response to pathogens, potentially offsetting the negative selective pressures from Celiac Disease (Hale 2017). *SORBS1* is associated with obesity and the development of type 1 and 2 diabetes (Lin et al. 2001; Germain et al. 2015), as well as reduced Tuberculosis bacterial growth (Agrawal et al. 2016). *LEPR* encoding the leptin receptor is another gene of interest, due to its association with obesity-related outcomes in modern individuals (Clement et al. 1998; Farooqi et al. 2007), as well as its involvement in the immune system (De Rosa et al. 2015). Importantly, *LEPR* has been reported as a selection candidate using both ancient and modern data (Voight et al. 2006; Colbran et al. 2021).

**Fig. 4.**
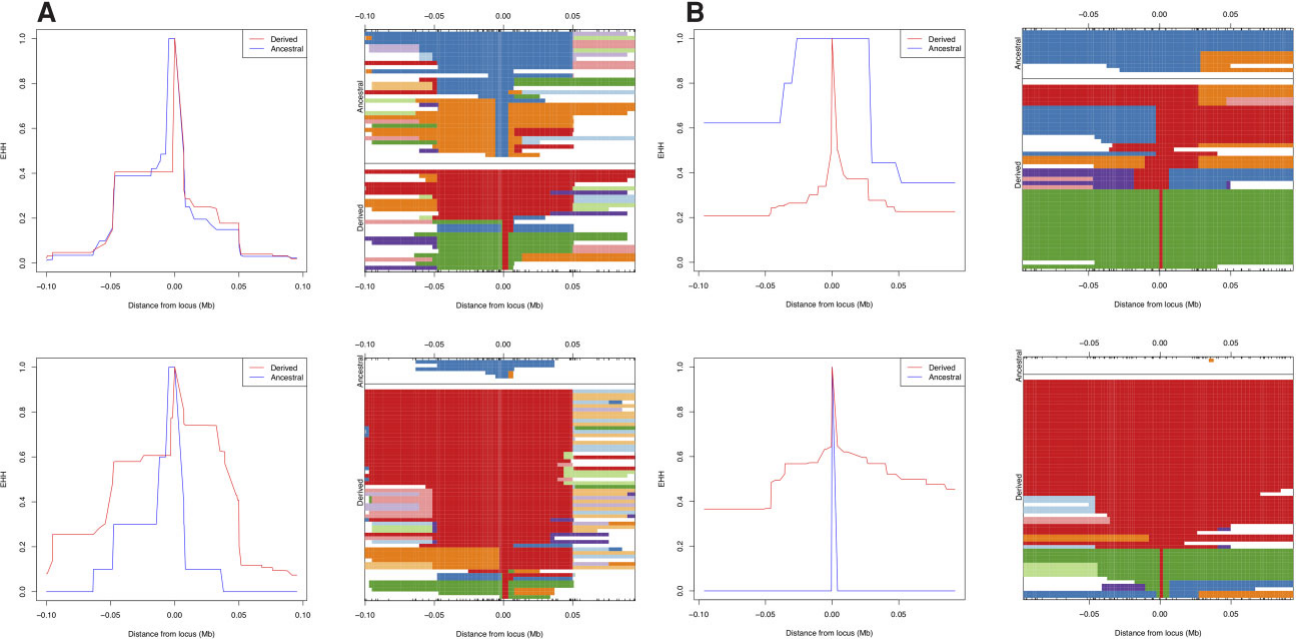
Extended haplotype homozygosity. (*A*) LMF SNP rs12933840, (*B*) JAK1 SNP rs3790541. Top panels show the EHH for the ancestral and the derived alleles for HG, and the bottom panels for LBK.

## Conclusions

On the basis of comparative analyses of genome-wide genetic variation at ∼1.24M SNPs in EEF, we find similar ancestry proportions at Derenburg-Meerenstieg II, as we see in other LBK sites across Germany and the broader LBK distribution zone (Hofmanová et al. 2016; Lipson et al. 2017; Rivollat et al. 2020). Overall, we find that LBK individuals closely resemble western Anatolian early Neolithic farmers, suggesting a rapid population expansion from western Anatolia to Central Europe. Moreover, the results from our LBK individuals suggest that there was no, or very limited, admixture between the local European HG and the incoming farmers during the pioneer phase of the early Neolithic in Central Europe. We show that all EF belonged to a relatively large source population compared to the local HGs, and found no signal of recent inbreeding, which means that larger population sizes were initially maintained. The relative genetic similarities between EFs allowed us to group them together, leveraging the increased cohort size for selection scans. Despite there being a limited difference in time between the LBK and Anatolian Neolithic individuals, and potential ongoing gene flow from Anatolia and southeastern Europe, the overlapping results of the three selection scans we explored and of the HLA class-I and II analysis suggest that Early Neolithic individuals in central Europe were experiencing an ongoing metabolic and immune function adaptation to the Neolithic lifeways as a result of the increased disease burden in group sizes that exceeded those of HGs. Moreover, we see an adaptation to higher latitudes in Neolithic individuals compared with modern Africans and HGs, as evidenced by allele frequency changes of variants associated with pigmentation and folate synthesis. The Neolithization has been considered one of the strongest natural selective forces acting on the human genome. Our results show that there are multiple signals of selection that can be observed in the LBK and the EFs in general in association with the transition to an agricultural lifestyle and sedentism. However, our findings also highlight the challenges of studying selection using aDNA, especially in light of complex admixture or demographic histories and limited time that elapsed since the split of populations under study. We also stress the need for increased cohort sizes and refinement of statistical tools that can take these confounding factors into account.

## Materials and Methods

### Archaeological Background/Site Description

Located in the shadow of the Harz (Hercynian) mountain range, the amount of precipitation in the area around DER in the Mittelelbe-Saale region is just under 530 nm/a and the loess-shaped slope area is covered by thick layers of topsoil, in some cases up to 1 m, which has favored the preservation of skeletal material. The archaeological site was excavated between 1997 and 1999, and represents a continuous settlement from the Early (LBK) and Middle Neolithic (Rössen, Ammensleben) to Bronze and Iron Age (Fritsch et al. 2011). The LBK-associated graveyard represents a closed find and encompassed 43 graves, as well as two separate graves outside the graveyard. A total of 32 single grave, one double, one triple burial, two burials in settlement pits, two burials with secondary inhumations, and one empty grave were found. The majority of DER individuals were buried in East-West orientation in a varying flexed position, which was a common practice in the Neolithic. Slightly more than half of the skeletons exhibited a very good to moderate degrees of preservation (grade 1–3 = 100–50% preserved from skeleton), whereas other burials were be poorly (grade 4 = 25–50%) or very poorly preserved (grade 5 = <25%) (Nicklisch 2017).

### Sample Preparation

Samples were processed using the following aDNA extraction protocol (Velsko et al. 2020), with the UDG-half treatment. Samples (*N* = 37) were first shotgun sequenced followed by the 1,240k capture on the samples (*N* = 32) with more than 0.1% endogenous DNA for select SNPs (supplementary table S1, Supplementary Material online). The raw fastq files were processed and mapped to the human reference hs37d5 with default parameters from EAGER (Peltzer et al. 2016). Minimum mapping and base qualities of 30 were used.

### Contamination and aDNA Authentication

aDNA status was authenticated using MapDamage2.0 (Jonsson et al. 2013). Two base pairs were trimmed from the UDG-half bam files following the aDNA damage pattern using trimBam as implemented in BamUtil 1.0.13 (Jun et al. 2015). X-chromosome contamination estimation in males was performed using ANGSD (Korneliussen et al. 2014) following the software manual. First step: “angsd -r X:5000000-154900000 -doCounts 1 -iCounts 1 -minMapQ 30 -minQ 30”, second step: “contamination -a angsdCounts -h HapMapChrX.gz 2 > out”. Contamination estimates from samples with more than ∼200 SNPs on the X chromosome covered were considered as reliable (supplementary table S1, Supplementary Material online). Mitochondrial DNA contamination was estimated using Schmutzi (Renaud et al. 2015) and contamMix (Fu et al. 2013).

### Genotyping

Pseudohaploid genotyping was performed using SAMtools mpileup (Li and Durbin 2009) and PileupCaller (https://github.com/stschiff/sequenceTools/blob/master/src/SequenceTools/PileupCaller.hs) for the 1,240k sites. Pseudohaploid genotypes were merged with the Human Origin Affymetrix dataset of modern individuals around the globe (Patterson et al. 2012), as well as published ancient West Eurasian individuals (https://reich.hms.harvard.edu/allen-ancient-dna-resource-aadr-downloadable-genotypes-present-day-and-ancient-dna-data, version 44.3).

SmartPCA from EIGENSOFT v7.2.1 (http://www.hsph.harvard.edu/alkes-price/software/) was used to perform a PCA projection of ancient samples on top of the variation of the modern data. Unsupervised admixture was performed on k components from 1 to 17 with random seeding after LD pruning in plink with the following settings (-indep-pair-wise 200 25 0.4). Cross-validation (CV) values were compared, and the *k* component 11 with the lowest CV error was selected.

### *F*-statistics

R package *admixr* was used to perform f-statistics and *qpAdm* (Petr et al. 2019). We performed f4 tests to determine if there were differences in HG ancestry in the individuals from DER compared with other EEF groups, as well as to test whether LBK individuals had additional HG ancestry compared to individuals from Anatolia Neolithic (Bar8 and Bar31 (Hofmanová et al. 2016)). Based on the admixture analysis, we formally tested the admixture components using *qpAdm* as implemented in the *admixr* package (Petr et al. 2019) using a 12 sample outgroup (Mbuti, Papuan, Onge, Han, Karitiana, Mota, Ust-Ishim, Russia_Mal'ta1, Vestonice, CHG, Israel_Natufian, Villabruna) + additional European HG sources (Villabruna, Russia_EHG, GoyetQ2). The admixture event between WHG and Anatolian farmers was determined using DATES v. 753 (Moorjani et al. 2016).

### Biological Relatedness and ROH Analyses

READ (Monroy Kuhn et al. 2018) and lcMLkin (Lipatov et al. 2015) were used to perform tests of biological relatedness. ROH analysis was performed using HapROH on samples with more than 600k SNPs based on the 1,240k capture (Ringbauer et al. 2020).

### Mitochondrial and Y Haplotype Analysis

To determine mitochondrial DNA haplogroups, bam files were mapped to the mtDNA rCRS sequence (Andrews et al. 1999). Haplogrep 2.0 was used to assign mitochondrial haplogroups. Y-haplotype analysis was performed using the method described in (Rohrlach et al. 2021).

### Shotgun Data

Three individuals (DER002, DER009, BDB001) were shotgun sequenced (PE75) to high coverage.

### HLA Typing

A development version of OptiType 2 (Szolek et al. 2014) was used to determine HLA Class I and II alleles from ≥30 bp single-end sequencing data retrieved from the immunocapture libraries (https://github.com/FRED-2/OptiType, tag DER). Fifteen of 432 allele calls were overruled in favor of runner-up alleles based on anomalous coverage patterns induced by short reads cross-mapping with alleles of different loci.

### Selection Scans

LSBL was used on the 1,240k genotype data to determine sites in the genome that have undergone recent selective sweeps. This method relies on pair-wise Fst values for three populations to identify loci with extreme Fst difference between population of interest and two other groups used for comparison. Resulting LSBL values were ranked and empirical *P* values were determined. Resulting SNPs were annotated using the R package BioMart (Durinck et al. 2009).

We also performed a pathway enrichment analysis using gowinda (Kofler and Schlotterer 2012) comparing the top 1% LSBL hits to all loci analyzed (1,240k SNPs after QC). This method performs gene-set enrichment base on GO classifications and designed specifically for GWA studies. Gowinda was designed to take into account the assumptions that are often violated in genome-wide associations studies, such as longer genes having more SNPs, and overlapping genes being sampled in clusters (Kofler and Schlotterer 2012). Gowinda performs internal permutations by randomly sampling SNPs from the total set of SNPs. By recording the associated genes and performing the permutation multiple times, gowinda establishes an empirical null distribution, and the significant overrepresentation of candidate SNPs is determined compared with the empirical null distribution (Kofler and Schlotterer 2012).

### AIMLESS Admixture-Informed Maximum-Likelihood Estimation for Selection Scans

For site i on the genome, we calculate the allele frequency for two source populations and a target population, denoted $f_{i}^{S_{1}}$, $f_{i}^{S_{2}}$ and $f_{i}^{T}$, respectively. We then calculate the optimal value of the mixing parameter ${\hat{\gamma }}_{i}\hat{o}[0,1]\;$ such that we minimize the value of the function

$$f(\gamma ,f_{i}^{T},f_{i}^{S_{1}},f_{i}^{S_{2}})=(\;f_{i}^{T}-[\gamma \;f_{i}^{S_{1}}+(1-\gamma )f_{i}^{S_{2}}]{)}^{2},$$

and define the constrained allele frequency estimator to be ${\hat{\;f}}_{i}^{T}=\hat{\gamma }$_i_$\;f_{i}^{S_{1}}+(1-\hat{\gamma }i)f_{i}^{S_{2}}$. Note that if $f_{i}^{S_{1}}=f_{i}^{S_{2}}$, then we simply let ${\hat{\gamma }}_{i}=1$.

We then calculate log-likelihood values for the unconstrained model and the constrained model assuming that $X_{i}∼B(N_{i},{\hat{\;f}}_{i}^{T})$ for the constrained model, and that $X_{i}∼B(N_{i},f_{i})$ for the unconstrained model, denoted *l*_1_ and *l*_0_. Naturally, as *f*_*i*_ is the maximum-likelihood estimator for a binomial observation, *l*_0_ ≥ *l*_1_.

Finally, we perform a LRT, with test statistic Λ_*i*_ = −2(*l*_1_ − *l*_0_), where$\;\Lambda_{i}∼X_{2}^{2}$. Via a simulation study based on the observed allele frequencies in Anatolia_N and WHG populations, we identified a *P* value cutoff of 1 × 10^−8^ that returned a false discovery rate of zero, with the highest positive discovery rate.

Note that we filter sites for which we have observed allele counts from at least 10 individuals from each population, and for which the allele frequency in the source populations is >zero.

### Haplotype-Based Scan

Genome-wide 1,240k capture data from Derenburg, LBK and Iron Gates HG individuals were imputed using GLIMPSE following the standard procedures (Rubinacci et al. 2021). Genotype likelihoods were computed using bcftools mpileup (Li 2011). The GLIMPSE_phase function was used to run imputation in chunks with window size of 2,000,000 bps and buffer size of 200,000 bps. The chunks were then ligated using GLIMPSE_ligate and haplotypes were sampled using GLIMPSE_sample. The 1000G reference panel was used for imputation (The 1000 Genomes Project Consortium 2015). Samples with endogenous DNA coverage on the 1,240k capture of above 0.5× were included in the analysis. REHH package was used to run haplotype-based selection scans (Gautier and Vitalis 2012).

## Supplementary Material

msac108_Supplementary_Data

## Data Availability

Genomic data (BAM and fastq format) are available at the European Nucleotide Archive under accession number PRJEB52488, genotypes in eigenstrat format can be found at https://edmond.mpdl.mpg.de/dataset.xhtml?persistentId=doi:10.17617/3.HOKI5I The code for AIMLESS is available at https://edmond.mpdl.mpg.de/dataset.xhtml?persistentId=doi:10.17617/3.HOKI5I.
