# Supplementary material for: Population Genetics and Signatures of Selection in Early Neolithic European Farmers
Source: Mol Biol Evol. 2022 May 17;39(6):msac108. doi: 10.1093/molbev/msac108 (PMC9171004; doi:10.1093/molbev/msac108)
Supplement: msac108_Supplementary_Data [file msac108_supplementary_data.zip › Supplementary_Figures_FINAL.docx]

**SUPPLEMENTARY FIGURES**

**Supplementary Figure 1. F4 statistics in the form f4(Mbuti, HG; test, Anatolia_N_Bar).** Models with the absolute Z-score > 3 are shown in orange, all other models are shown in grey.

**Supplementary Figure 2. F4 statistics in the form f4(Mbuti, test; Loschbour, IronGates).**

**Supplementary Figure 3. DATES output for Derenburg individuals**

**Supplementary Figure 4. Patrilocality estimates.** Individuals are plotted from the lowest mean pairwise mismatch rate to the highest. Dashed lines represent the mean values for adult and sub-adult males and females.

**Supplementary Figure 5**. Allele counts for select SNPs associated with phenotypic traits.

**Supplementary Figure 6. PCA with the imputed samples that have been used in the selection scan. Samples are colored by cohort.** Samples are projected onto modern human variation using smartPCA. Imputed and non-imputed pseudohalpoid samples are plotted.

**
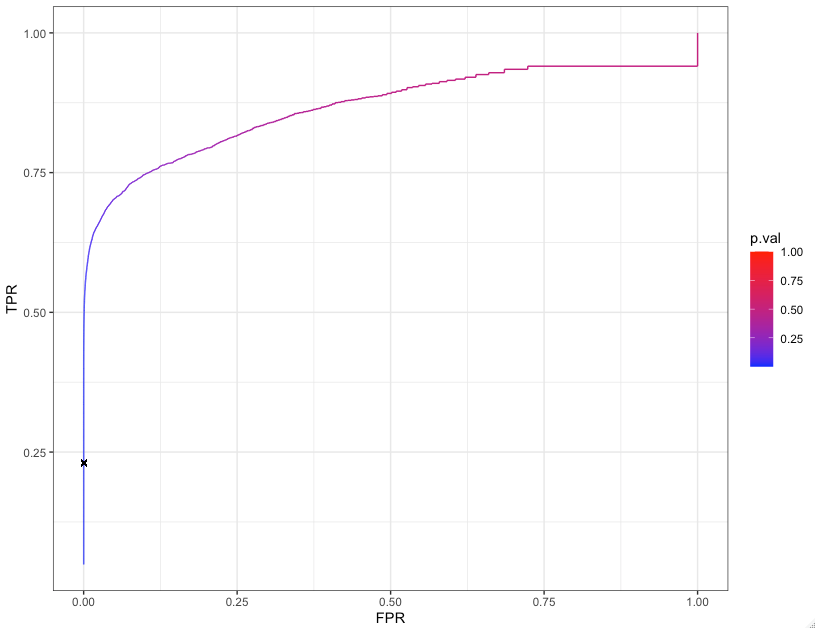
**

**Supplementary Figure 7. ROC curve for AIMLESS.** The false and true positive rates for
*p*=1×10^−8^ marked with an x.


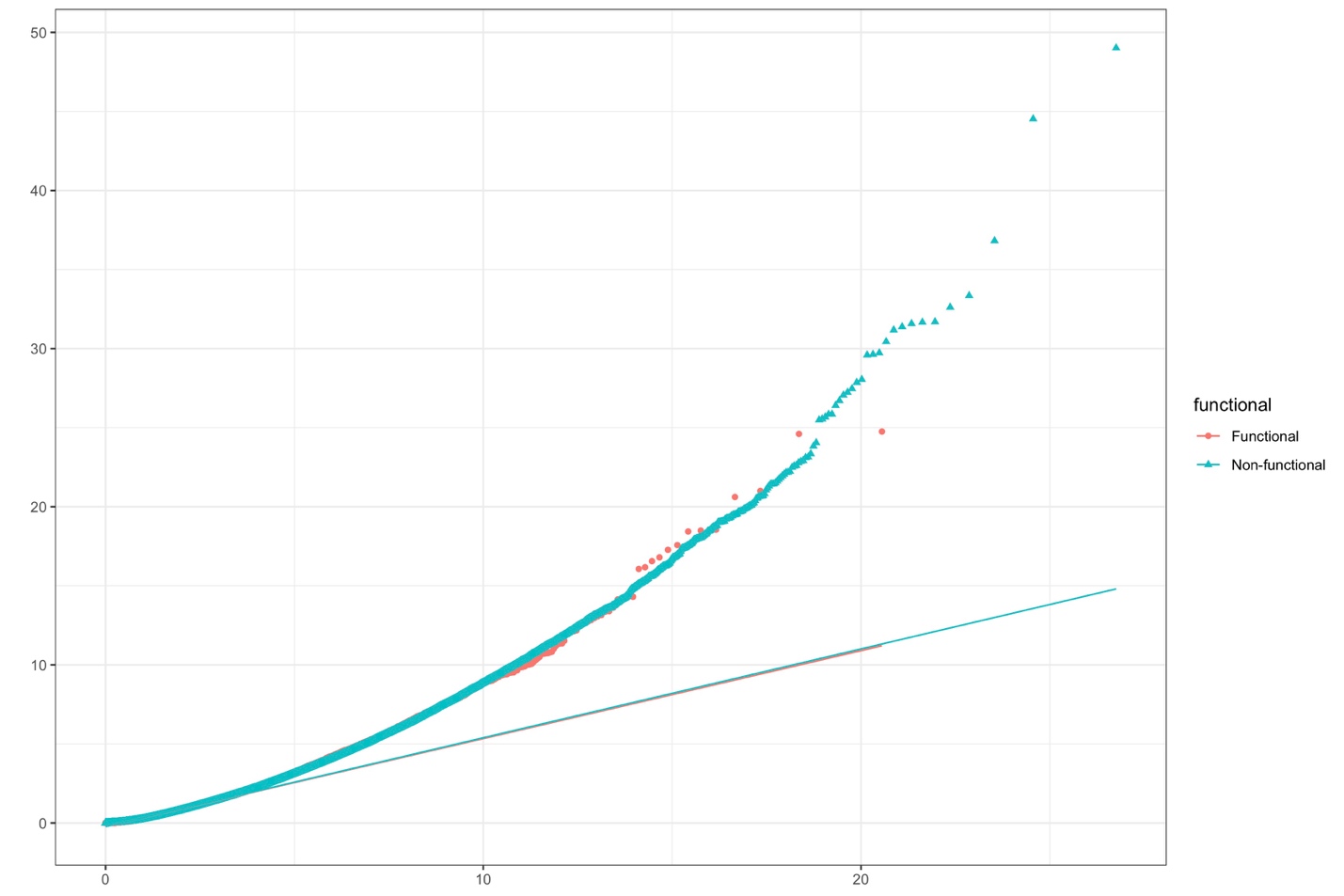


**Supplementary Figure 8. QQplot of expected and observed LRTs from AIMLESS. Functional and non-functional SNPs are indicated with different colors.**

B

A

**Supplementary Figure 9. Imputation down-sampling experiments with Stuttgart Mühlhausen.** (A) genotype agreement between the diploid and the down-sampled data, (B) percent homozygosity.

**Supplementary Figure 10. Revigo TreeMap based on the significant GO terms (FDR < 0.05) from gowinda.** A single cluster is represented by a rectangle. Clusters are joined into ‘superclusters’ of loosely related terms and represented with different colors. The size of the rectangle reflects the -log10(p-value) of the GO term.

**Supplementary Figure 11. Skin color adaptation selection signal in *SLC24A5.*** The top panels show the EHH distribution among the derived and the ancestral alleles in the Iron gates hunter gatherers, while the lower panels are focused on the LBK. The focal SNP in both cases is rs1426654.

**Supplementary Figure 12. Metabolic adaptation selection signal in *FADS1.*** The top panels show the EHH distribution among the derived and the ancestral alleles in the Iron gates hunter gatherers, while the lower panels are focused on the LBK. The focal SNP in both cases is rs174546.
